# Aggregation in particle rich environments: a textural study of examples from volcanic eruptions, meteorite impacts, and fluidized bed processing

**DOI:** 10.1007/s00445-018-1207-3

**Published:** 2018-03-02

**Authors:** Sebastian B. Mueller, Ulrich Kueppers, Matthew S. Huber, Kai-Uwe Hess, Gisela Poesges, Bernhard Ruthensteiner, Donald B. Dingwell

**Affiliations:** 1 Ludwig-Maximilians-Universität München LMU, Munich, Germany; 2 University of the Free State, Bloemfontein, South Africa; 3 Vrije Universiteit Brussel, Brussels, Belgium; 4 Ries Krater Museum Nördlingen, Nördlingen, Germany; 5 Zoologische Staatssammlung München, Munich, Germany

**Keywords:** Particle aggregation, Volcanic ash, Meteorite impact, Granulation

## Abstract

Aggregation is a common process occurring in many diverse particulate gas mixtures (e.g. those derived from explosive volcanic eruptions, meteorite impact events, and fluid bed processing). It results from the collision and sticking of particles suspended in turbulent gas/air. To date, there is no generalized model of the underlying physical processes. Here, we investigate aggregates from 18 natural deposits (16 volcanic deposits and two meteorite impact deposits) as well as aggregates produced experimentally via fluidized bed techniques. All aggregates were analyzed for their size, internal structuring, and constituent particle size distribution. Commonalities and differences between the aggregate types are then used to infer salient features of the aggregation process. Average core to rim ratios of internally structured aggregates (accretionary lapilli) is found to be similar for artificial and volcanic aggregates but up to an order of magnitude different than impact-related aggregates. Rim structures of artificial and volcanic aggregates appear to be physically similar (single, sub-spherical, regularly-shaped rims) whereas impact-related aggregates more often show multiple or irregularly shaped rims. The particle size distributions (PSDs) of all three aggregate types are similar (< 200 μm). This proves that in all three environments, aggregation occurs under broadly similar conditions despite the significant differences in source conditions (particle volume fraction, particle size distribution, particle composition, temperature), residence times, plume conditions (e.g., humidity and temperature), and dynamics of fallout and deposition. Impact-generated and volcanic aggregates share many similarities, and in some cases may be indistinguishable without their stratigraphic context.

**Electronic supplementary material:**

The online version of this article (10.1007/s00445-018-1207-3) contains supplementary material, which is available to authorized users.

## Introduction

Particle aggregation is a common feature in nature and technology. It has been observed in the deposits of volcanic eruptions (e.g., Self 1983; Hayakawa 1990; Schumacher and Schmincke 1991; De Rita et al. 2002; Branney et al. 2008; Brown et al. 2010, 2012; Van Eaton and Wilson 2013; Scolamacchia and Dingwell 2014) and meteorite impacts (e.g., Graup 1981; Alegret et al. 2005; Pinto and Warme 2008; Cannon et al. 2010; Rocholl et al. 2011; Branney and Brown 2011; Huber and Koeberl 2017) and occurs during the processing of industrial powders (e.g., Salman et al. 2006). Several phenomena promote the aggregation of suspended particles, including (1) electrostatic forces (e.g., James et al. 2002; Bonadonna et al. 2011; Del Bello et al. 2015), (2) liquid bonding or freezing (e.g., Tomita et al. 1985; Gilbert and Lane 1994; Bonadonna et al. 2002; Durant et al. 2009; Van Eaton et al. 2012; Mueller et al. 2016), and (3) the growth of secondary mineral phases (Sheridan and Wohletz 1983; Gilbert and Lane 1994; Brown et al. 2010; Scolamacchia and Dingwell 2014; Mueller et al. 2016). Although the same underlying physical and chemical phenomena may occur in all environments under consideration here (volcanic eruptions, meteorite impacts, experiments), the aggregate P-T growth conditions, growth rates, and preservation potential are likely to differ significantly depending on their petrogenesis.

Throughout this paper, we describe the different sample types in the following order: (a) volcanic, (b) meteorite, and (c) experimental.

- Volcanic ash aggregates

Explosive volcanic eruptions can inject large volumes of volcanic ash into the atmosphere. Under certain circumstances, ash can cluster together and form aggregates, which have been documented in many deposits (e.g., Van Eaton and Wilson 2013; Wallace et al. 2013; Scolamacchia and Dingwell 2014), and there is growing evidence that aggregation is the norm rather than the exception (e.g., Brown et al. 2012). Observations of aggregate fallout indicate that many aggregates do not survive transport and/or sedimentation (e.g., Taddeucci et al. 2011; Bonadonna et al. 2011; Bagheri et al. 2016; Mueller et al. 2017a), and this has also been inferred from modeling and observations of secondary thickness maxima in ash deposits (Durant et al. 2009). Larger and strongly bonded aggregates, such as accretionary lapilli, do survive transport and deposition processes and are commonly preserved in deposits as, for example, in deposits of the eruptions of Tungurahua volcano, Ecuador (Kueppers et al. 2016), Soufriere Hills volcano, Montserrat (Burns et al. 2017), and Volcán de Colima, Mexico (Reyes-Dávila et al. 2016), as well as many others (e.g., Brown et al. 2012).

Volcanic ash aggregation strongly influences ash dispersal because ash aggregates have different aerodynamic properties to their constituent ash particles (e.g., Durant et al. 2009). This can result in premature fallout from the atmosphere (e.g., Sisson 1995; Durant et al. 2009) and changes to the proximal and distal ash mass loadings in the eruption plume. Based on aggregate analysis from recent eruptions together with experimental and numerical studies, a better mechanistic and quantitative understanding of the parameters relevant inside an eruption plume is emerging (Costa et al. 2010; Van Eaton et al. 2012; Del Bello et al. 2015; Mueller et al. 2016). As a result, ash aggregation has been incorporated in ash-plume forecasting models (Folch et al. 2016).

(b) Meteorite impact events

Meteorite impact events produce large dust-rich ejecta clouds (e.g., French and Koeberl 2010). Particle aggregates have been documented in impact deposits on Earth, Mars (Fralick et al. 2012), and Moon (McKay et al. 1970), where they have been used as impact event indicators. On Earth, aggregates have been described in numerous meteorite impact deposits, such as those from the Sudbury (1850 ± 1 Ma; Ontario, Canada; Cannon et al. 2010; Huber and Koeberl 2017), Stac Fada (1199 ± 70 Ma, Scotland; Branney and Brown 2011), Alamo (382 ± 4 Ma; Nevada, USA; Pinto and Warme 2008), Chicxulub (66 ± 0.3 Ma; Gulf of Mexico; Alegret et al. 2005), and Nördlinger Ries (14.94 ± 0.07 Ma; Germany; Graup 1981; Rocholl et al. 2011) impact events.

The basic process of impact cratering is reasonably well understood (e.g., Melosh 1989; French and Koeberl 2010): upon impact, fragmented target rock is accelerated away from the impact site as an excavation flow, leaving a crater. The accelerated particle flow emerges above the surface, ejects material ballistically and produces an expanding material cone, the ejecta curtain. Most of the material is deposited within a few crater radii; however, a small fraction is ejected significantly further and accordingly deposited at greater distances. Glassy ejecta bombs from the Nördlinger Ries meteorite impact crater, for example, are found 1000 km away (Gentner 1971; Koeberl 1992, Schwarz and Lippolt 2002). Several models have been proposed to explain impact-related particle aggregates. Johnson and Melosh (2014) suggest that aggregates form within the ejecta curtain. A similar conclusion of aggregation of silicate particles from a cooling ejecta plume was reached for distal (about 1000 km) aggregates from the Chicxulub impact crater by Yancey and Guillemette (2008). Alternatively, distal aggregates have been attributed to deposits from particulate density currents, physically similar to volcanic pyroclastic density currents (e.g., Addison et al. 2010; Branney and Brown 2011). Grieve et al. (2010) examined the Onaping Formation in the Sudbury crater, which represents fallback material within the crater itself, and concluded that the aggregates formed as a result of melt-fuel coolant interaction (MFCI): water flowed into the crater causing phreatomagmatic eruptions, allowing aggregates to form over several generations, and examples are shown of broken aggregates that have been coated with ash and incorporated into other aggregates. Accretionary lapilli in the Nördlinger Ries impact crater have been attributed to the fallback of melt-rich impact breccia (which forms the characteristic suevite upon deposition, Graup 1981) and the gravitational collapse of unlithified suevitic breccias (Stöffler et al. 2013).

(c) Artificial aggregation

Aggregation is a key process in the industrial processing of many powders (e.g., food, feed, pharmaceutical, fertilizer, detergent, and mineral powders). Powders are typically processed under controlled laboratory settings in fluidized beds. For this purpose, gas is fluxed from below through initially stagnant particles. At sufficient flux, particles are lifted and set in a chaotic motion, following stochastic streamlines (Salman et al. 2006). Aggregate formation in fluidized beds can be described in terms of population balance, which describes temporal changes of particle property distributions. Particle size enlargement through aggregation is controlled by operating conditions such as moisture, initial grain-size distribution, processing time, pneumatics, and/or thermal conditions (Smith and Nienow 1983; Banks and Aulton 1991; Watano et al. 1996; Iveson et al. 1998; Turton et al. 1999; Uhlemann and Mörl 2000). Under these controlled boundary conditions, the dependence of aggregation efficiency and preservation from the input parameters can be constrained empirically (Mueller et al. 2016).

A structural and textural classification scheme for volcanic ash aggregates has been proposed by Brown et al. (2012), which can also be applied for impact and artificial aggregates. Aggregates are divided into two main categories: particle clusters (PC) and accretionary pellets (AP). Particle clusters can further be sub-classified into ash clusters (PC1) and coated particles (PC2). Accretionary pellets are sub-divided into poorly structured pellets (AP1), pellets with concentric structure (AP2), and liquid pellets (AP3). Based on this classification scheme, we analyze volcanic, impact, and artificial aggregates, examine the textural differences and commonalities, and determine the resulting implications for generation mechanisms.

## Methodology

Natural samples were collected during field studies and then transported to the lab, analyzed, and classified. Three methods were applied to unravel the inner structure of aggregates: (a) cutting and grinding, (b) thin section analysis, or (c) X-ray computed tomography (CT) of the samples. CT has the advantage of being non-destructive and was used for the rare impact aggregate samples.

The granulometry of artificial aggregates and of several volcanic aggregate samples was determined using a Coulter® LS230 laser diffraction particle size analyzer (Fraunhofer optical model, imaginary/real refractive indices of 0.001/1.52 for glass beads and 0.1/1.52 for volcanic ash). All artificial aggregate and several volcanic aggregate samples were weak enough to be disintegrated into single particles in an ultra-sonic bath which were then measured with the LS230. For strongly cemented volcanic aggregate and impact aggregate samples that could not be disintegrated in the ultra-sonic bath, thin section analysis- and computed tomography (X-ray CT)-based data have been used to evaluate size ranges of single particles using ImageJ software (Schneider et al. 2012).

We apply the aggregate classification scheme of Brown et al. (2012) to volcanic, artificial, and impact aggregate samples. We also present special types of aggregates that do not fit in this classification scheme.

### Sample overview

- Volcanic ash aggregates

Caldera del Rey tuff ring, Tenerife, Canary Islands.

Caldera del Rey is a 1.13 million-year-old (Huertas et al. 2002) phonolitic tuff ring complex (Paradas-Herrero and Fernandez-Santin 1984). Ash aggregates were sampled from a sequence of cm- to dm-thick beds of primarily massive and cross-bedded tuffs that contain occasional pumice lapilli clasts and that show significant lateral thickness variations (Table 1). Ash aggregates are abundant (< 10–40 vol%, Brown et al. 2010). Aggregates typically become more abundant and clast-supported towards the top of each bed. No apparent change in aggregate size, structure, or abundance could be observed throughout the sampled stratigraphy.

**Table 1:** Overview of volcanic aggregate samples. Listed are sample latitudes, longitudes, ages, stratigraphic units, their distances to the postulated eruptive vent, and the types of aggregates (PC1, PC2, AP1, AP2) that were found in respective sample locations

Sample location	Latitude	Longitude	Age	Stratigraphic unit	Distance of sample to postulated vent (km)	Ash clusters (PC1)	Coated particles (PC2)	Poorly structured pellets (AP1)	Pellets with concentric structure (AP2)
Caldera del Rey, Tenerife^a^	28° 04′ 19.23″ N	16° 43′ 08.99″ W	1.13 Ma	PDC	0.1	No	Yes	No	Yes
Secche di Lazzaro, Stromboli^b^	38° 46′ 32.72″ N	15° 12′ 23.30″ E	5–13 ka	Fallout	2	Yes	Yes	Yes	Yes
Monte Razzano, Sabatini Volcanic District^c^	42° 07′ 45.46″ N	12° 22′ 24.04″ E	90 ka	Fallout	1.6	Yes	Yes	No	No
Baccano Caldera, Sabatini Volcanic District^d^	42° 06′ 25.38″ N	12° 22′ 29.02″ W	90 ka	Fallout	0.1	Yes	No	No	No
Lago di Martignano, Sabatini Volcanic District^e^	42° 06′ 13.23″ N	12° 19′ 00.76″ E	90 ka	PDC	0.05	Yes	No	Yes	Yes
Stracciacappa, Sabatini Volcanic District^f^	42° 08′ 08.13″ N	12° 19′ 05.14″ E	90 ka	PDC	0.3	Yes	No	Yes	Yes
Cave, Colli Albani^g^	41° 48′ 52.52″ N	12° 55′ 43.56″ E	600 ka	PDC	18	No	No	Yes	Yes
Empiglione, Colli Albani^h^	41° 57′ 07.42″ N	12° 51′ 31.79″ E	600 ka	PDC	25	No	No	Yes	Yes
Valle Lungherina, Colli Albani^i^	41° 55′ 52.71″ N	12° 50′ 22.20″ E	600 ka	PDC	20	No	No	Yes	Yes
Solfatara, Campi Flegrei^j^	40°49′41.63” N	14°08′19.35″ E	6.2 ka	Fallout	0.1	Yes	No	Yes	No
Montserrat, Soufrière Hills^k^	16°43′15.21” N	62°08′59.95” W	2010 AD	Fallout	5.4	No	No	Yes	Yes
Nickenich, Eifel^l^	50° 24′ 41.13″ N	07° 20′ 21.41″ E	12.9 ka	co-PDC Fallout	5	Yes	Yes	Yes	No
Wehrer Kessel Volcano, Eifel^m^	50° 24′ 44.95″ N	07° 13′ 56.31″ E	12.9 ka	Fallout	2	Yes	No	No	No
Tungurahua^n^	01° 25′ 53.98″ S	78° 27′ 20.46″ W	2006 AD	PDC	4.5	No	Yes	Yes	Yes
Santo Antão^o^	17° 05′ 35.06″ N	25° 12′ 33.83″ W	100 ka	PDC	5.3	Yes	No	No	No
Masaya Caldera^p^	11° 47′ 58.07″ N	86° 16′ 41.83″ W	33.8 ka	Fallout and PDC	23.6	No	No	Yes	Yes

^a^Spain (Huertas et al. 2002); ^b^Italy (Giordano et al. 2008); ^c–f^Italy (De Rita et al. 1994; Sottili et al. 2010); ^g–i^Italy (De Rita et al. 1995a, b); ^j^Italy (Di Vito et al. 1999; Isaia et al. 2009, 2015); ^k^Montserrat, West Indies (Stinton et al. 2014); ^l–m^Germany (Schmincke 2009; Förster and Sirocko 2016); ^n^Ecuador (Douillet et al. 2013); ^o^Cape Verde (Holm et al. 2006); ^p^Nicaragua (Bice 1985; Freundt et al. 2010)

### Stromboli Volcano, Eolian Islands, Italy

Ash aggregates were collected from the ~ 5 ka Secche di Lazzaro succession (SDL) at Punta Lena (Giordano et al. 2008), on the SW coast of Stromboli (Table 1). The SDL succession resulted from some northward-directed collapse that allowed seawater to come in contact with magma in the upper part of the plumbing system and triggered a phreatomagmatic eruption. Our samples come from a clast-supported, matrix-free lens of aggregates that sit above the lower SDL unit (UA, see Fig. 7a, Giordano et al. 2008) which consists of aggregate-rich, thinly bedded ash tuffs, deposited from dilute pyroclastic density currents (PDCs). It contains aggregates up to 5 mm in diameter and is interpreted as a fall deposit.

### Monte Razzano, Baccano Caldera, Lago di Martignano and Stracciacappa, Sabatini Volcanic District, Italy

The Sabatini Volcanic District (SVD) is part of the potassic Quaternary Roman Province and extends over an area of 1800 km^2^, including the city of Rome (De Rita et al. 1994). Monte Razzano and Baccano Caldera are both part of the Baccano Eruptive Center (BEC) within the SVD. The Baccano pyroclastic succession is dominated by massive to variably laminated ash-lapilli tuffs with subordinate ash or lapilli fallout horizons (Sottili et al. 2010). Aggregates sampled at Monte Razzano occur in a poorly sorted, matrix-supported ash-lapilli tuff with low aggregate content (Table 1). Aggregates at Baccano Caldera occur within a fine-grained ash tuff with subordinate pumice and lithic lapilli. Lago di Martignano is an 86 ka composite maar located east of Bracciano lake (Sottili et al. 2010). Samples were collected inside the caldera from a poorly sorted, cross-bedded PDC deposit (Table 1). Stracciacappa is 97 ka hydromagmatic center (maar) north of Lago di Martignano (Sottili et al. 2010). Samples come from laminated and cross-bedded lapilli-tuffs of PDC origin in the northern crater wall (Table 1).

### Colli Albani, Italy

The large composite caldera complex of Colli Albani (Alban Hills) is located about 30 km SE of the city of Rome, Italy (De Rita et al. 1995a). Three locations at the foot of the Apennines were chosen for sampling: (1) the Valle Lungherina valley and the villages of Empiglione and Cave (Table 1). At all sites, the sampled deposits were well-sorted massive ash tuffs interpreted as fall deposits from co-PDC plumes (De Rita et al. 1995a, b).

### Solfatara volcano, Campi Flegrei, Italy

Solfatara is a 4.2 ka maar-diatreme volcano cut into older volcanic deposits. Eruptions were dominated by a series of explosions of variable intensity (Di Vito et al. 1999; Isaia et al. 2009, 2015). Aggregates were collected from well-sorted, strongly hydrothermally altered fall deposits exposed in the NNW crater wall.

### Soufrière Hills volcano, Montserrat, West Indies

Aggregates were sampled from the co-PDC fall deposits of the 11 February 2010 eruption (see Stinton et al. 2014; Burns et al. 2017). The sampled deposit consisted of a 10-cm-thick layer entirely composed of accretionary pellets. It is underlain and overlain by PDC deposits.

### Eifel volcanic field, Germany

The Eifel volcanic field in West Germany is characterized by numerous explosion craters and maars. Aggregates were sampled from the deposits of the Plinian phase of the 12.9 ka Laacher See eruption (Schmincke 2009, Table 1). These deposits are fine- to medium-grained, well-sorted ash beds and were deposited from ash clouds overriding PDCs from Plinian plume collapse (Schmincke 2009). At a separate location 3 km NW of Laacher See, aggregates were found within 150 ka deposits of an eruption at the Wehrer Kessel volcano (Förster and Sirocko 2016). Aggregates were sampled from an ~ 15-cm thick-, 3-m-long lens composed entirely of ash aggregates.

### Tungurahua volcano, Ecuador

Ash aggregates were collected from the deposits of the July–August 2006 eruptions. The August eruptions generated PDCs that flowed down the NW- and W-flank of the volcano (Douillet et al. 2013). Aggregates were observed in a fines-depleted lapilli tuff at the top of a several meter-thick deposit located where PDCs deposits had temporarily dammed the river (Kueppers et al. 2016).

### Santo Antão, Cape Verde

Aggregates were sampled on the island of Santo Antão, Cape Verde, which was dominated by two partly co-existing magmatic series of mafic shield-building phases until about 100 ka ago (Holm et al. 2006). Towards the end of the shield-building phase, highly explosive eruptions emplaced the Cão Grande Formation. Aggregates were sampled in the uppermost part of the dry river bed of the Ribeira do Canudo on the western plateau of Santo Antão. They occur in a single horizon intercalated with tephra-phonolitic PDC deposits from the initial phase of the Cão Grande 2 eruption, which overlies the phonolitic Cão Grande 1 pumice-fall deposit.

### Masaya Caldera, Nicaragua

Aggregates were sampled from an ~ 34 ka ignimbrite (Bice 1985) in a quarry at Canteras, about 7 km WSW of Diriamba. The scoria-rich ignimbrite belongs to the upper part of the Pleistocene Las Sierras Formation which was erupted from a caldera adjacent to Masaya Caldera (Freundt et al. 2010). The ignimbrite is composed of three depositional units 1–3 (Freundt et al. 2010). Units 1 and 2 likely to represent the same eruption, while unit 3 followed after a significant time interval of several years. Aggregates occur in units 1 and 3, in both fall and PDC deposits.

(b) Impact Aggregates

### Nördlinger Ries impact crater, Germany

The 15 Ma old, 25-km-diameter Nördlinger Ries impact crater (Rocholl et al. 2011; Stoeffler et al. 2013) was created by the impact of a 1.1–1.5-km-diameter asteroid (Artemieva et al. 2013). The target stratigraphy comprised 600–700 m of Triassic and Jurassic sedimentary rocks overlying crystalline basement (Pohl et al. 1977; Hüttner and Schmidt-Kaler 1999; Stoeffler et al. 2002). The crater volume estimates range between 124 and 200 km^3^ (Pohl et al. 1977; Hörz et al. 1983; von Engelhardt and Graup 1984). Immediately after impact, a primary ejecta curtain started emplacing primary suevite—a widespread layer of massive, very poorly sorted, clast- to matrix-supported fallback sediments with abundant clasts of molten ejecta—directly on top of parautochthounous (as uplifted) crystalline basement (Artemieva et al. 2013; Stoeffler et al. 2013). Later ejecta deposits in and outside the crater are finer-grained and lack melt clasts (Graup 1981). Thirteen boreholes have been drilled inside the morphologic crater, ten of which penetrated the suevite and reached the underlying crystalline basement (Stoeffler et al. 2013). Accretionary lapilli occur in suevite units in three boreholes. Samples in this study come from the Nördlingen 1973 drillhole (FBN 73) at depths of 296 and 301 m. In all three cases, the aggregates are found only in the transition zone between post-eruptive lake sediments and underlying suevite, where they are sparsely distributed within fine grained (< mm) layers. This has been interpreted as fallout from the uppermost part of the primary ejecta curtain, rich in solid particles and water vapor. Aggregates in the Deiningen and the 1001 drillholes have been described elsewhere (Mosebach 1964; Förstner 1967; Graup 1981).

### Sudbury impact structure, Canada

Sampled Sudbury aggregates are derived from the Connors Creek location (see Huber et al. 2014). The deposit comprises ~ 1 m of lithic (chert) breccia, overlain by ~ 1 m of breccia that also contains melt glass and accretionary lapilli. Cannon et al. (2010) include an overlying ~5 m thick layer that grades from brecciated material at the base to finer material near the top, but note that the accretionary lapilli are found in beds that are cross-bedded, with thin bands of fine-grained breccia between the beds, which may be evidence of reworking.

(c) Artificial aggregates

### Glatt Ingenieurtechnik GmbH, Weimar, Germany

Artificial aggregates used in this study were produced via fluidized bed techniques at Glatt Ingenieurtechnik GmbH, Weimar, Germany, from soda-lime glass beads (< 50, 40–70, and < 70 μm) and natural volcanic ash (< 40, 40–90, and < 90 μm) collected from deposits of the Laacher See eruption (Germany). Grain-size data is provided in the Supplementary Dataset. Mueller et al. (2016) produced both particle clusters (PC) and accretionary pellets (AP) under controlled and reproducible conditions. The following parameters were varied during the experiments to examine their influence on aggregation: (1) initial particle size distribution, (2) humidity, (3) viscosity of the liquid binder, (4) Reynolds numbers of the fluidized particles, (5) gas velocity, (6) salt concentration on the particle surfaces, (7) temperature, and (8) processing time. Aggregates were produced through spraying either a NaCl-bearing solution of H_2_O or a 37% HCl solution. In both cases, the liquid phase induces aggregation following collisions, and the resultant salts cement the particles together after evaporation of the liquid. Salts (mainly NaCl) are either generated through re-crystallization upon H_2_O evaporation, or through chemical reactions between the HCl phase and the glass (Mueller et al. 2017b). The aggregates produced in this manner are between 0.5–5 mm in size, depending on experimental parameters (Mueller et al. 2016).

## Results: structural analysis

- Volcanic ash aggregates

**Fig. 1:**
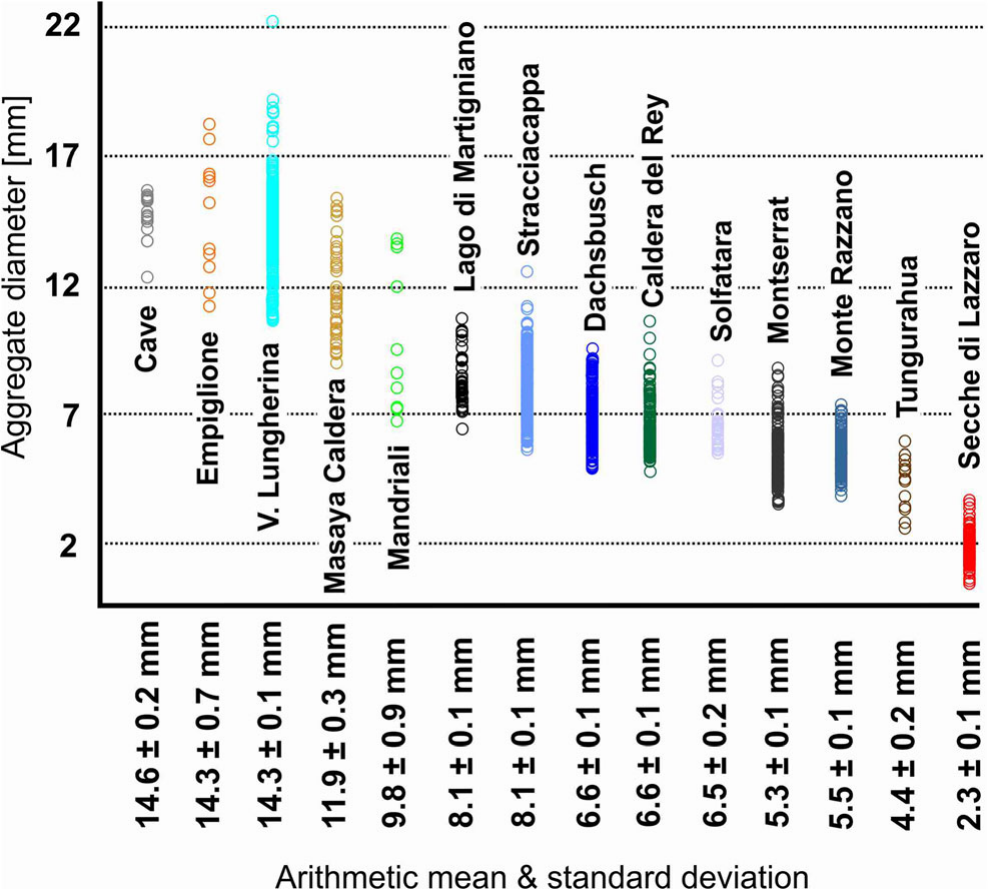
Size ranges of more than 1100 sampled volcanic aggregates. Mean size (diameter) and standard deviation are shown. Size ranges of aggregates are clearly confined to several mm

Samples analyzed in this study represent mainly PC and AP aggregate types (plus all subcategories except mud drops, AP3). In total, more than 1100 volcanic aggregates were analyzed. Aggregate sizes range from 1 mm (e.g., Secche di Lazzaro, SDL) to 2 cm in diameter (e.g. Valle Lungherina, Fig. 1 & Supplementary Dataset). In this study, aggregates from the same sample location generally exhibit restricted size ranges (Fig. 1); this contrasts with other studies where a wide range of aggregate sizes exist at a single location (e.g., Wallace et al. 2013).

**Fig. 2:**
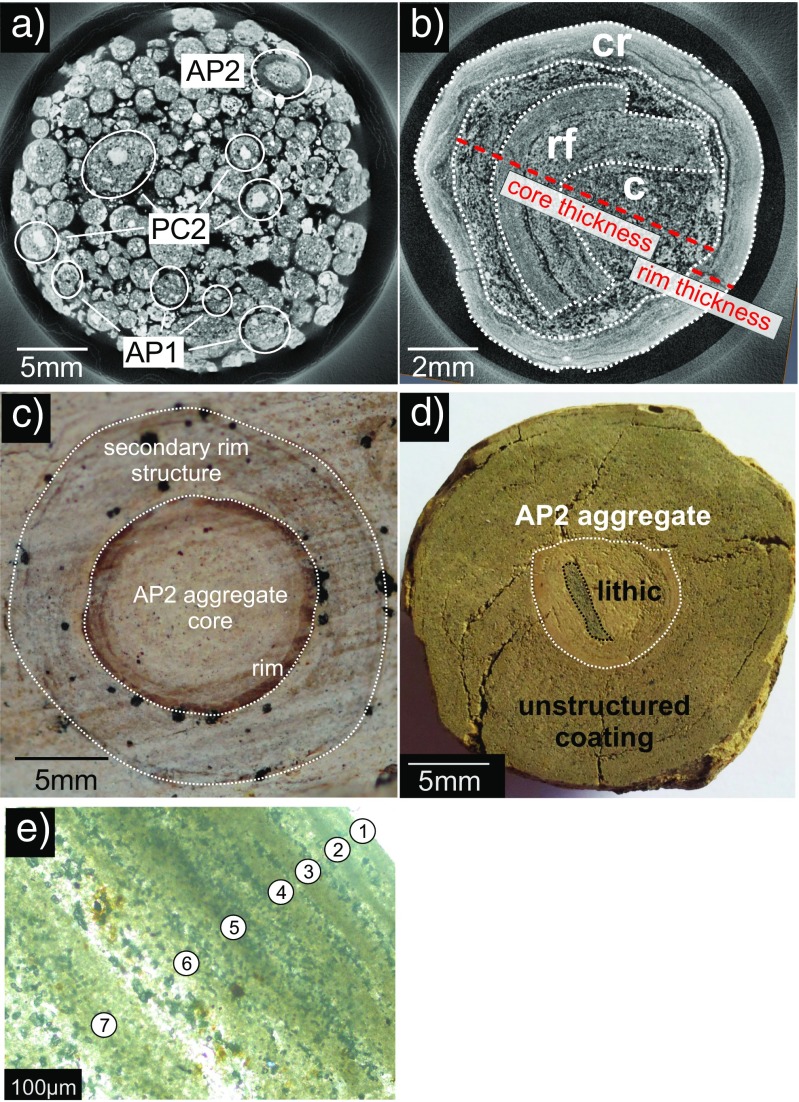
Details of various samples at various resolutions. **a** X-ray CT analysis of a 2.5-cm-wide drill core from the Secche di Lazzaro succession on Stromboli. The drill core contains PC1, PC2, and AP2 type aggregates. Most aggregates represent the PC1 type. **b** AP2 similar type aggregate from Empiglione, Italy, 10 mm across. The X-ray image shows a broken rim fragment (rf) being recycled to act as a core (c) of a new accretionary pellet coated by a concentric rim structure (cr). **c** sample from Caldera del Rey, Tenerife, showing a “double” accretionary pellet: a small, inner accretionary pellet acts as a core for an even larger accretionary pellet. Concentric structures typical for APs are existent in both the inner and the outer rim structures. The whole structure is 20 mm across. The dotted lines indicate the physical and optical separation of the inner accretionary lapilli from the outer rim. **d** Accretionary pellet sample from Masaya Caldera, Nicaragua. The inner, spherical accretionary pellet (AP2) has a black lithic fragment acting as a core (“armored pellet”). Compared to the Caldera del Rey sample (Fig. 2c), there are no concentric rim features in the outer part of the sample existing. **e** Close-up of the rim of an AP2 sample of Valle Lungherina, exhibiting the repetitive formation of rims with a grain size fining outwards

Diverse types of aggregates were found very close to each other, sometimes within the same stratigraphic unit. For example, in the SDL succession on Stromboli (Table 1), a single drill core (2.5-cm diameter, 5 cm long), analyzed by X-ray CT, revealed lithic fragments coated with thick ash layers resembling to some extent PC2 type aggregates, poorly structured pellets (AP1), and pellets with concentric structure (AP2, Fig. 2a). Mixed populations of aggregates were also found at Caldera del Rey (see also Table 1).

Besides aggregates that fall into the PC/AP classification of Brown et al. (2012), we also sampled aggregates that can be described as coated accretionary pellets: a hybrid between PC2 and AP2 type aggregates. These aggregates are spherical, cm-sized pellets with concentric rim structures, exhibiting cores composed of either broken fragments of other aggregates (e.g., the rims fragments in the samples from Empiglione, Fig. 2b) or lithic fragments (aggregates at Masaya Caldera, Fig. 2c). Other samples can be described as double featured, i.e., accretionary pellets that have a second accretionary pellet as a core (Fig. 2d). Rarely, accretionary pellets show an accumulation of several thin (10 s of μm), concentric rims around a central core, such as in the Valle Lungherina sample (VL) where up to seven distinct rims are present (Fig. 2)e.

**Fig. 3:**
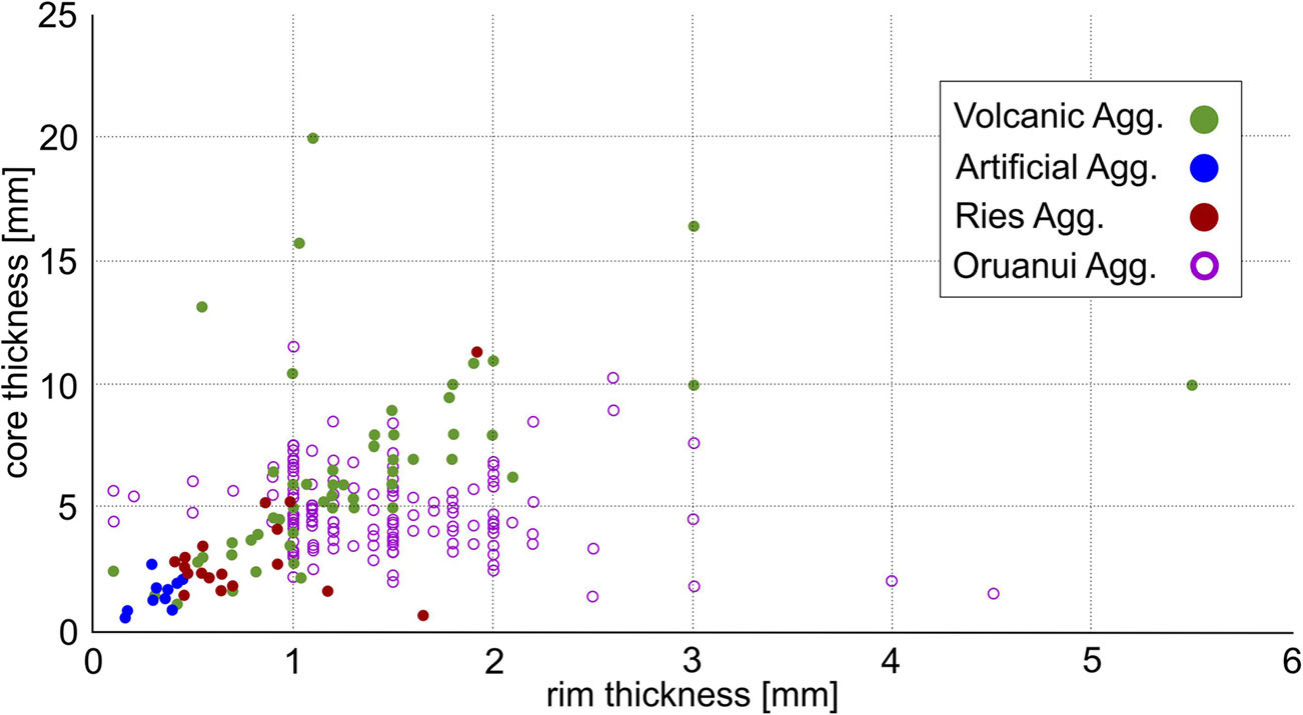
Measured core vs. rim thicknesses for volcanic, artificial and Noerdlinger Ries samples. Additional data for comparison from Van Eaton and Wilson (2013) for aggregates from the 25.4 kya Oruanui eruption from Taupo volcano, New Zealand, have been added and show good agreement with our data. Some Ries and Oruanui samples show clearly increased rim thicknesses compared to the average data plot

To quantitatively compare the structure of volcanic aggregates, core and rim diameters of > 100 AP type aggregates were measured with a digital caliper (Fig. 3). Core and rim diameters (see also Fig. 2b for measurement procedures) average 6.0 ± 0.5 and 1.32 ± 0.1 mm, respectively, with a mean core to rim ratio for volcanic aggregate samples of 4.54 ± 0.13 (Fig. 3 & Supplementary Dataset). For comparison, we plot data from Van Eaton and Wilson (2013) from the 25.4 ka Oruanui eruption of Taupo volcano, New Zealand. Some of these samples show very thick rims compared to their cores, but the overall core to rim ratio is smaller (average at 3.41) compared to our volcanic samples. This seems to indicate a slightly different aggregation pattern, allowing the rims to grow for a longer period or at very efficient rates.

The structural characteristics show that aggregation is a complex process that may see several phases of aggregation and aggregate breakup (Mueller et al. 2017a). Aggregation may initiate around larger clasts (coated lapilli, Fig. 2b, d) or through the growth of ash pellets (Fig. 2a, c) with variable grain size and sorting. Certain processes may allow aggregates to become mechanically strong (by, for example, cementation through the precipitation of secondary mineral phases and possibly also by freezing). Fragments of such hard aggregates can form sites for new aggregates (Fig. 2b).

(b) Meteorite impact aggregates

**Fig. 4:**
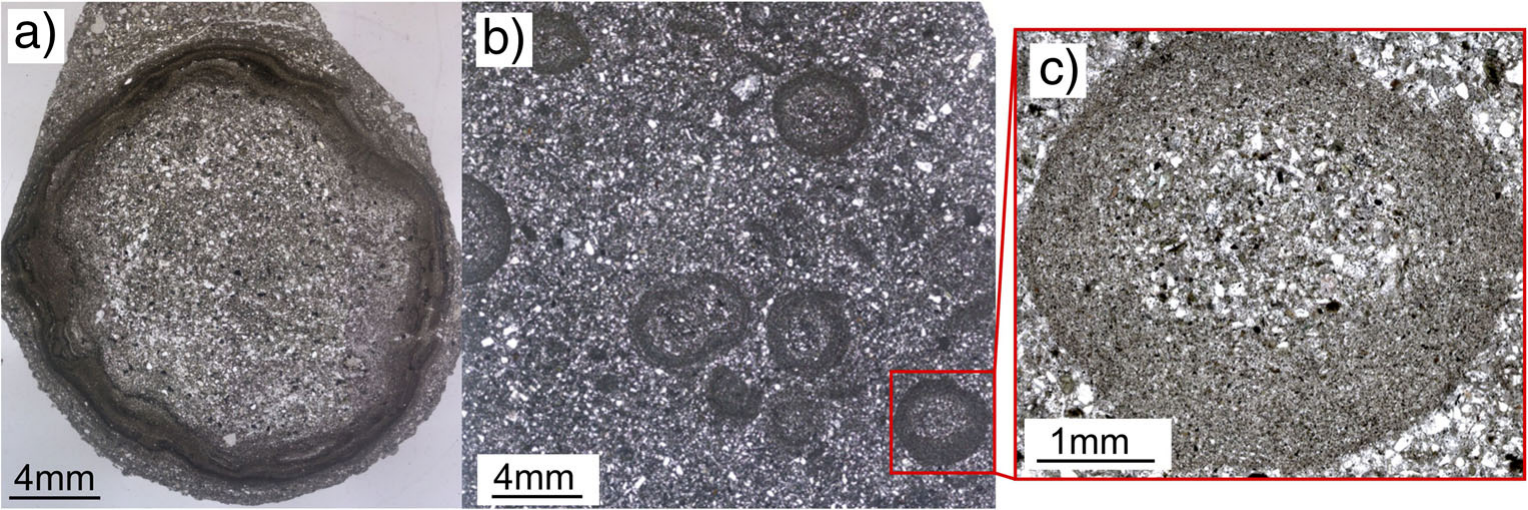
**a** Accretionary pellet sample from the Nördlingen 1973 drill hole (FBN 73) inside the Nördlinger Ries, Germany, from a depth of 296 m. Sample shows a clear separation into core (approx. 80% of the volume) and rim based on PSD. The overall shape of the pellet is spherical, but a wavy pattern of variably thick “growth rims” can be observed. **b** Shows a thin section also of the FBN 73 drill hole and a depth of 309.5 m. **c** Matrix supported accretionary pellets are spherical in their shape and show rims with thicknesses equalizing the according core thickness

The 19 analyzed samples from drill cores from the Nördlinger Ries impact crater consist exclusively of AP1 and AP2 aggregates. Aggregates range in size from 2 to 10 mm and have spherical shapes. In contrast to the volcanic ash aggregates, some of the impact aggregates have very irregular, wavy rim structures (Fig. 4a). The mean core and rim thicknesses are 3.2 ± 0.5 and 0.78 ± 0.1 mm, respectively, and their mean core to rim ratio of 4.1 ± 0.21 is generally comparable with volcanic samples. However, some aggregates have rim diameters that exceed the core diameters and show similarities with the Oruanui data (Figs. 3 and 4b, c Supplementary Dataset).

**Fig. 5:**
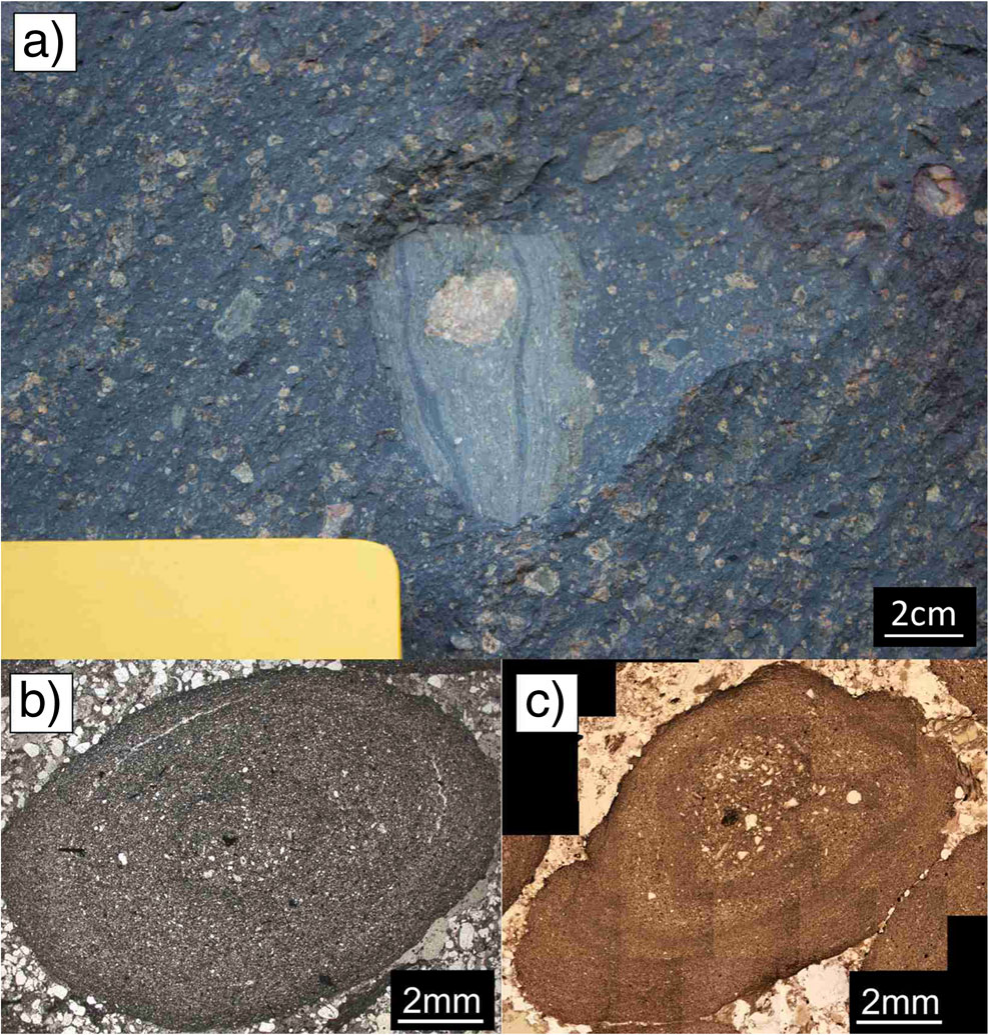
Accretionary features from Sudbury impact structure, Canada. **a** Lithic breccia (Onaping formation) containing accretionary lapilli in cross-bedded beds, intersected with fine-grained breccia. **b**, **c** Accretionary lapilli collected at Connors Creek site, some 450–500 km from the impact location. Lapilli consistently show coarse grained cores and finer grained rims, sometimes several concentric ones. The lapilli show different states of preservation, indicating variable degrees of shape alteration by mechanical erosion

Aggregates from the Sudbury impact deposits (Fig. 5a) consist of individual accretionary lapilli that are up to 2 cm in diameter. In cross-section, the lapilli exhibit multiple concentric rims. The lapillus imaged in Fig. 5b has a core of 4.5-mm diameter and an inner rim of 2.0 mm; the outer rim, present only around half of the lapillus, is 2.8 mm in diameter. The lapillus in Fig. 5c has a core of 4.0 mm, an inner rim of 2.6 mm, and an outer rim of 2.0 mm.

(c) Artificial aggregates

Structures of artificial aggregates are described briefly here; for a more detailed analysis, see Mueller et al. (2016). With the ProCell Lab®, we were able to reproduce both PC and AP aggregates. Depending on the initial particle size distribution (PSD), the resulting aggregates were either: (a) non-spherical and structureless (PC1), (b) spherical and structureless (AP1), or (c) spherical and internally structured (AP2). PC1 aggregates were produced with highly confined starting PSDs (< 40- or 40–90-μm particles only); AP1 aggregates appeared under the use of broader starting PSDs (i.e., < 90-μm particles) and fully evolved. AP2 aggregates were generated at starting PSDs of < 300-μm particles. Artificial aggregates are generally smaller than volcanic aggregates and exhibit diameters ranging between several 100 μm and a few mm, and have modes between 1 and 2 mm (depending on experimental conditions). The largest artificial aggregate diameters (5 mm) were achieved through the use of high viscosity binders (37% HCl) that sometimes allowed two or three APs to connect with each other and form aggregate clusters with diameters up to 1 cm. Mean core and rim thicknesses are 1.40 ± 0.20 and 0.33 ± 0.03 mm, respectively. Despite their small size, artificial aggregates show a similar core to rim ratio (4.24 ± 0.28, see Fig. 3 & Supplementary Dataset) to the volcanic ash aggregates.

## Results: textural analysis

- Volcanic ash aggregates

**Fig. 6:**
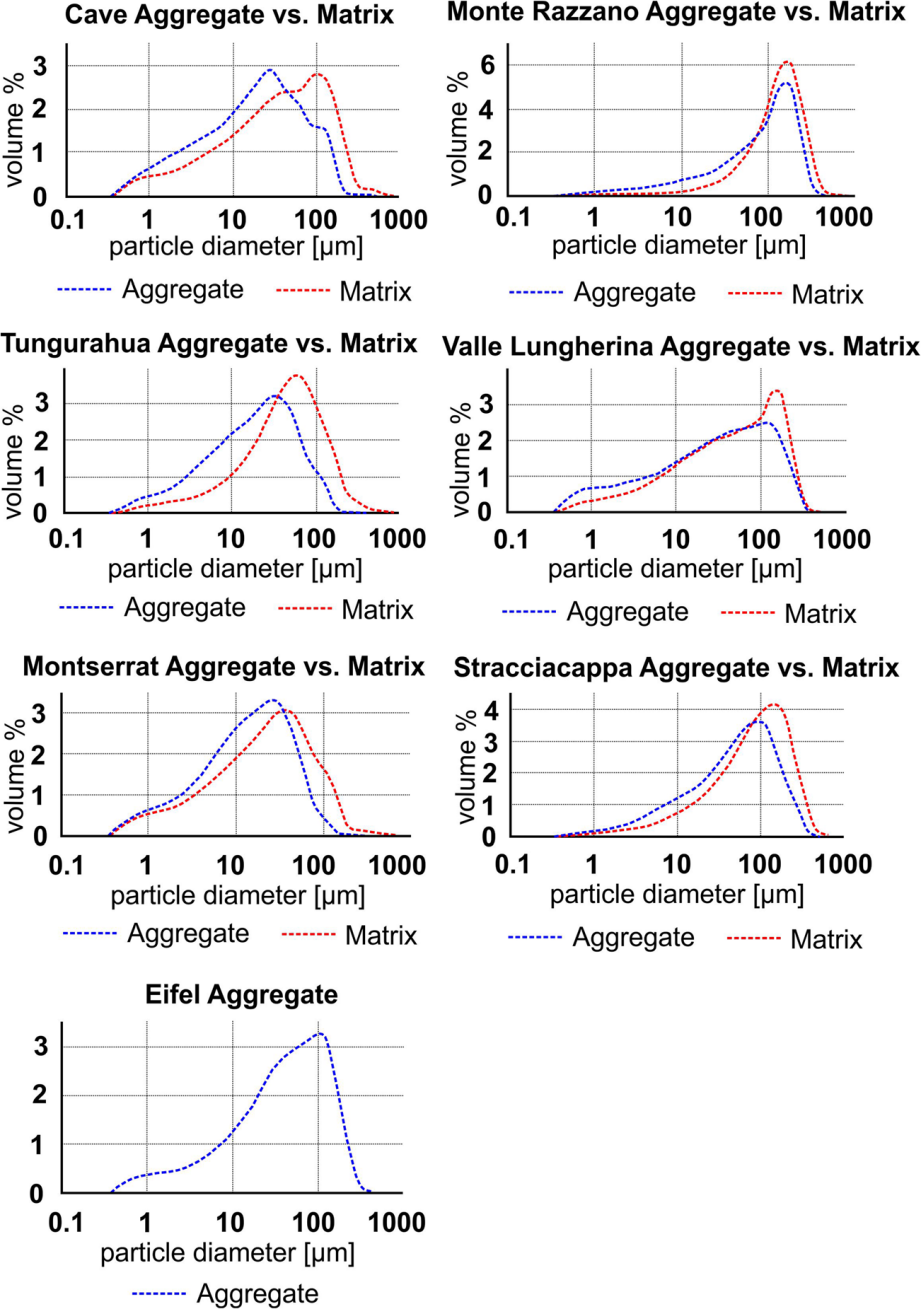
Particle size distribution (PSD) of aggregates vs. the surrounding matrix. PSD of aggregates (core and rim together) is systematically finer grained (modes between 20 μm (e.g., Cave) and 120 μm (e.g., Razzano)) compared to the surrounding matrix. For comparison, PSD of matrix < 250 μm only is shown

**Fig. 7:**
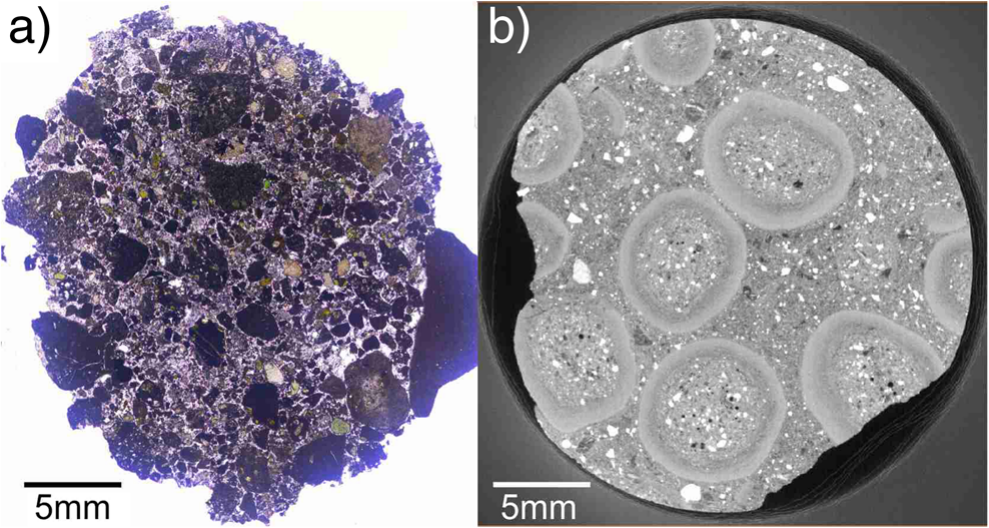
Examples of aggregates containing abundant coarse clasts. **a** Cape Verde particle cluster (PC) with primary particle sizes of up to several mm. **b** Drill core from Caldera del Rey, Tenerife, analyzed with X-ray CT. AP2 type aggregates are embedded in matrix material. AP2 aggregates have coarse-grained cores and fine-grained rims; PSD of aggregates does not exceed maxima of 500 μm. Matrix material lacks fine-grained ash as it is bound in the rims of the aggregates, and PSD does not exceed maxima of 1.5 mm

Grain size analysis by laser diffraction (Coulter® LS230) requires particles suspended in water. Samples from seven different locations (Valle Lungherina, Cave, Monte Razzano, Stracciacappa, Montserrat, Tungurahua, and Laacher See) could be disintegrated with minimal mechanical force. We determined the PSDs of up to ten aggregates from each location (Fig. 6). Samples show a typical particle size range of < 200 μm with their modes around 100 μm for Eifel, Stracciacappa, Monte Razzano, and Valle Lungherina and 20–50 μm for Tungurahua, Montserrat, and Cave. Maximum particle sizes in the volcanic aggregate samples were ~ 200 μm. X-ray CT and thin section analyses of samples that could not be disaggregated revealed much coarser-grained volcanic aggregates: for example, PC1 samples from Santo Antão contain clasts of up to several mm in diameter (Fig. 7a). AP2 samples from Tenerife contain 500-μm-diameter particles in their cores (Fig. 7b).

We sampled the surrounding unconsolidated matrix for aggregates extracted from PDC deposits. PDC deposits were matrix-supported, and maximum grain sizes of the matrix exceeded the maximum clast size of aggregates significantly (up to ten times, e.g., Tenerife, Fig. 7b). Aggregate rims were much finer in grain size than matrix material, e.g., Fig. 7b). Matrix material of six of the seven previously named sample locations has been sieved at 250 μm (~ 50–80 wt% of total deposit materials < 255 μm), which conforms to the maximum grain size generally observed in aggregates. All matrix materials except for Monte Razzano and Valle Lungherina, have coarser grained modes than their respective aggregates; all six sample locations reveal an enrichment of fine material (< 40–80 μm) in aggregate material compared to matrix material (Fig. 6 & Supplementary Dataset).

**Fig. 8:**
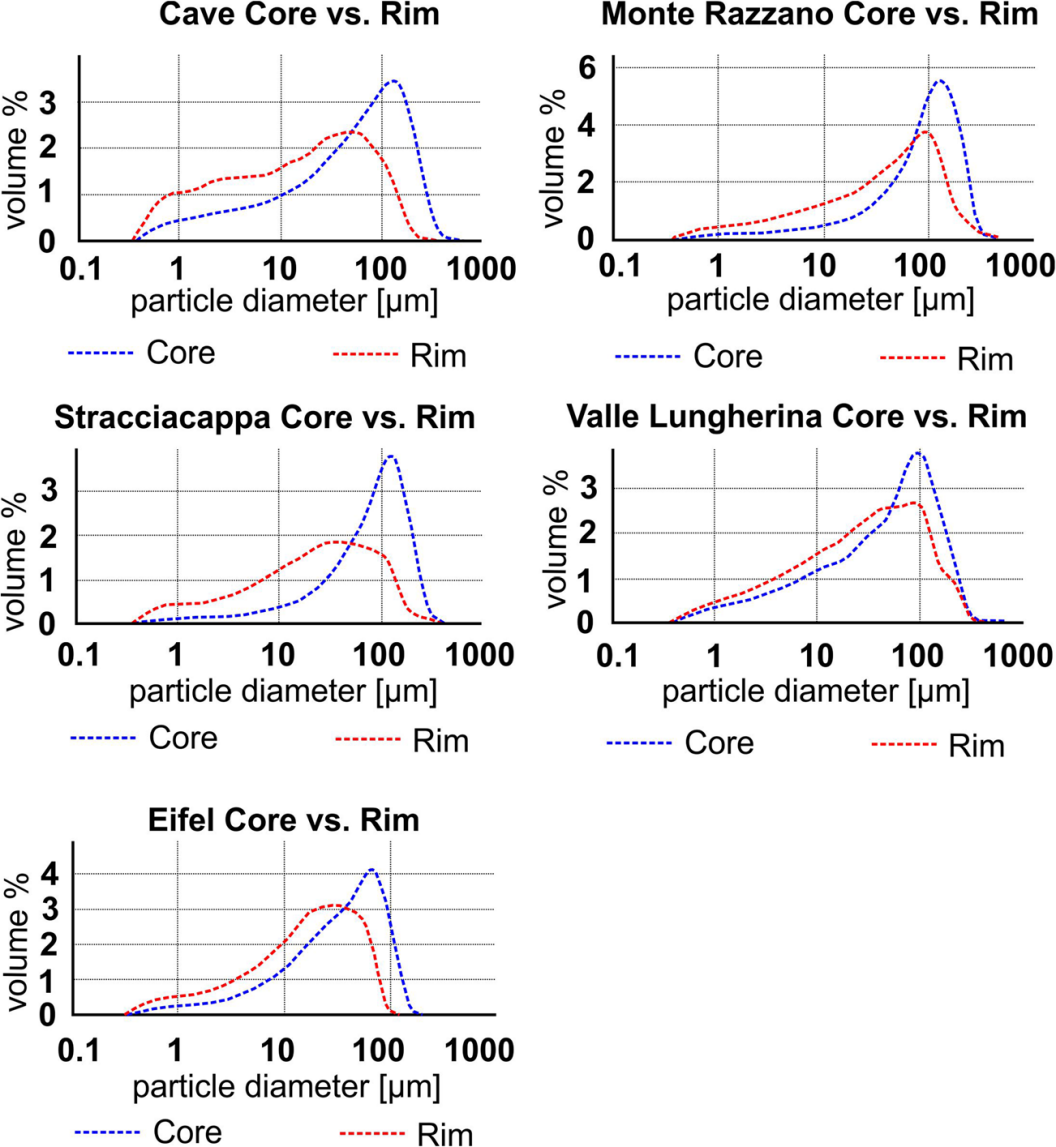
Particle size distribution (PSD) within aggregates: PSD of cores and rims has been measured and compared to each other: all cores show a coarser grained mode in PSD and have less fine material (< 60 μm) than the rims

For five locations (Valle Lungherina, Stracciacappa, Monte Razzano, Cave, and Eifel), it was possible to separate the rims from the cores of AP2 samples and measure them by laser diffraction (Fig. 8, Supplementary Dataset). All samples showed finer grained modes for their rim PSDs (40–100 μm) than for their core PSDs (110–140 μm): rims are enriched in fine material (< 60 μm) and cores are enriched in coarse material (> 60 μm).

(b) Meteorite impact aggregates

**Fig. 9:**
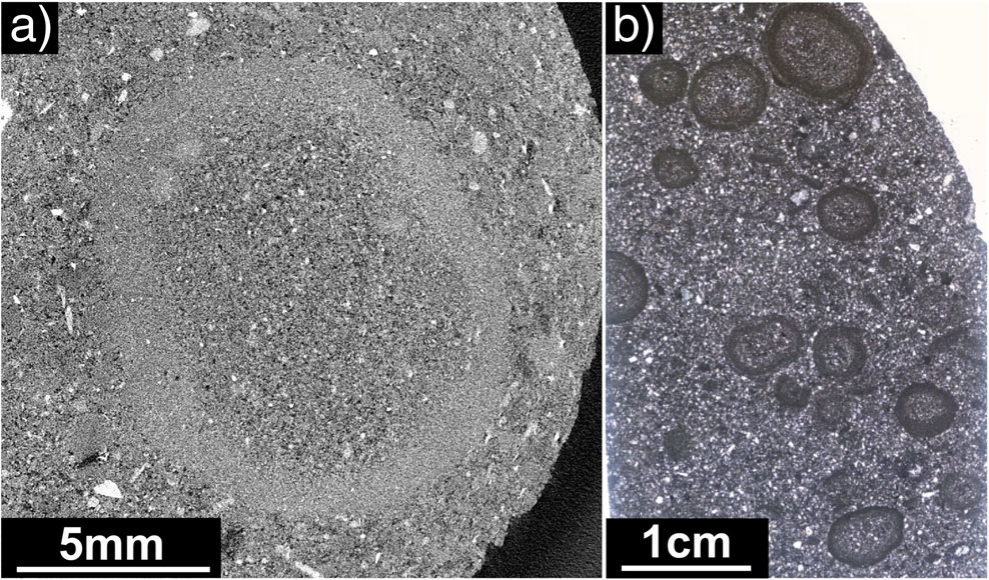
**a** X-ray CT image of an AP2 type sample from the FBN73 drill core inside the Nördlinger Ries, Germany, sampled at a depth of 296 m (see also Stoeffler et al. 2013). The aggregate is about 1.1 cm in diameter and has an overall PSD < 150 μm with a clearly finer PSD of about <85 μm in the rim. **b** Accumulation of AP2 type aggregates, also from the FBN73 drill core but sampled at a depth of 313 m. Aggregates are between 0.5 and 1 cm in size and show clear coarse-grained core and fine-grained rim features

It was not possible to analyze impact aggregates with the Coulter® LS230, because the aggregates are entirely lithified. Also, the spatial resolution of the X-ray CT was not sufficient to compute the PSD with Avizo, a software used to analyze CT data. Instead, ImageJ was used to estimate both the upper size range and average PSD of clasts building up the aggregates, based on thin sections and X-ray CT data. Particle size distributions smaller than approximately 100 μm were impossible to determine due to strong alteration of aggregate samples. Figure 9a shows an X-ray CT image of an AP2 type sample (found at a depth of 296 m) embedded in matrix. The sample is about 1.1 cm in diameter and has a PSD < 150 μm in its core and < 85 μm in its rim. The PSD mode is at about 100–130 μm for the core and 30–50 μm for the rim. Other analyzed aggregates, especially from the thin section shown in Fig. 9b, confirm these findings: the cores have a much coarser-grained peak in PSD than the rims.

(c) Artificial aggregates

**Fig. 10:**
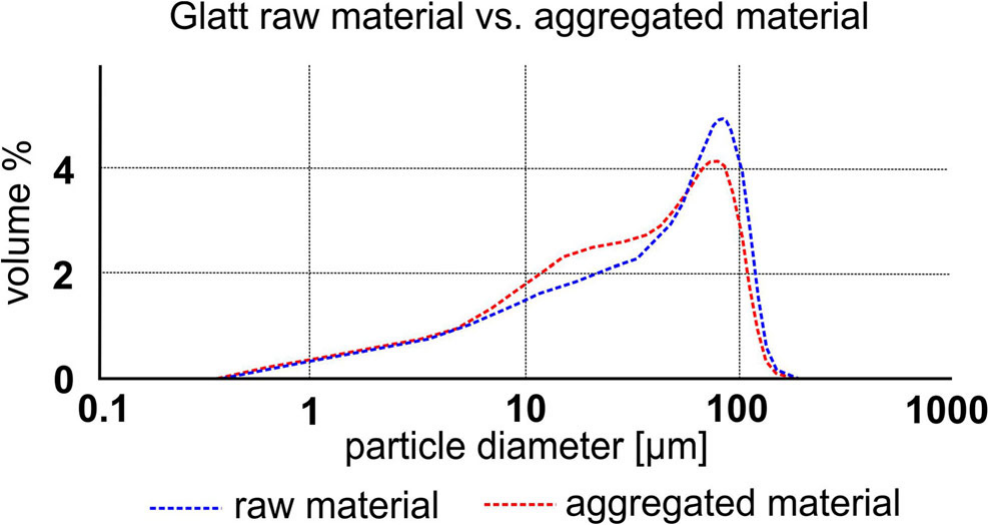
PSD of artificial aggregates produced with starting materials of nominally < 90 μm size. Enrichment of fines in aggregates, particularly between 5 and 60 μm, is visible. The aggregate dataset has been achieved by averaging more than 100 artificial aggregates

**Fig. 11:**
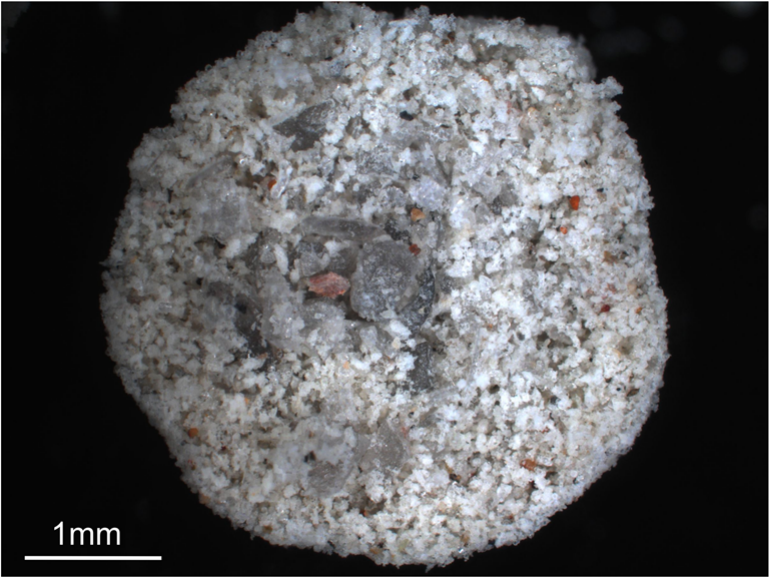
Artificial ash aggregate (AP2) produced of an initial PSD < 500 μm. Particles of several 100 s of μm are centered in the core region of the aggregate; the rim is built out of finer material. The aggregates show a heterogeneous distribution of particle packing of residual inter-clast pore space

Halite-cemented artificial aggregate samples were disintegrated in water and their PSDs measured with the Coulter® LS230. We analyzed 30 Laacher See ash aggregates that were generated with a fluidized bed of a PSD < 90 μm. These 30 measurements were averaged and compared to the mean PSD of the raw material (Fig. 10 & Supplementary Dataset). Aggregates and raw material share the same mode in their PSDs at 70 μm. However, the aggregates show a clear enrichment (approx. 10 vol%) of fine particles (< 50 μm) compared to the raw material. Artificial aggregation was not limited to materials < 90 μm. By increasing aggregation efficiency through the use of higher viscosity binders such as HCl (relative to H_2_O), we were able to generate AP2 aggregates out of a starting sample batch containing clasts as large as 500 μm. Because of the involved forces, aggregation is a size-selective process and large particles will be enriched in the cores (Fig. 11, Mueller et al. 2016).

## Interpretation

### Particle binding mechanisms

Large aggregates as described in this study are primarily generated through hydrostatic bonding forces and subsequent cementation. Several samples in this study are derived from dry eruptions (e.g., Tungurahua, Montserrat). Accordingly, the presence of accretionary lapilli in general, in pyroclastic deposits, should not be taken as a proxy for explosive eruptions involving external water at the fragmentation level (i.e., phreatomagmatic or Surtseyan). The geographic distribution of aggregate appearance in PDC deposits points to the strong influence of the ambient conditions. A free water phase (whether in the liquid or gaseous state) is omnipresent in volcanic eruption plumes, meteorite ejecta plumes, or ash clouds overriding PDCs. This water has several possible origins: magmatic origin (H_2_O is the most abundant volatile species dissolved in silicate melts, e.g. Mader 1998), external water from aquifers or surface water bodies (e.g., Edmonds and Herd 2005), or entrainment of ambient humidity from tropospheric air (e.g., Tomita et al. 1985). When hot PDCs travel across water bodies, significant volumes of steam can be added to the background humidity. In ash-rich environments, this may enable and/or enhance aggregation. Several of the investigated aggregates derive from deposits that show indications of such interactions as, for example, with a river (e.g., Tungurahua in 2006), lake (e.g., Sabatini Volcanic District), or ocean (e.g., Soufrière Hills Volcano, Montserrat 2010). Tomita et al. (1985) observed wet aggregates falling from the H_2_O-rich eruption plume of Sakurajima volcano, Japan and described high relative air humidity values of 85%.

Artificial aggregation experiments have shown that increasing the humidity in a particle-laden environment exponentially increases the aggregation rate (Mueller et al. 2016). Volcanic eruption plumes and impact ejecta curtains can entrain external humidity from the atmosphere or from, for example, the impactor target region (groundwater, lake, ocean, etc.) or the atmosphere. As particle aggregates bound by electrostatic forces have average diameters no larger than several tens of μm (James et al. 2002), we postulate that the generation mechanism of the cm-sized Nördlinger Ries aggregates involved hydrostatic bonding forces, coupled with subsequent cementation processes to stabilize them. Due to their age and alteration, it is not possible to analyze potential chemical binders within impact aggregates.

### Aggregate growth processes

The growth of aggregates is limited by physical forces: experiments have shown that aggregates will continue to grow for as long as the impact energies of colliding particles can be dissipated by the viscous forces of the liquid binding agent (Ennis et al. 1991). At a certain aggregate size, impact energies exceed the viscous dissipation forces of the liquid binder and cause the impacting particles to rebound. This halts aggregate growth (Mueller et al. 2016). This may explain the observation that aggregate populations are relatively homogeneous in size (Fig. 1). If critical aggregation parameters such as liquid binder viscosity and, as a consequence, liquid film thickness, PSD, humidity, or temperature do not change drastically, aggregates will stop growing once they have reached a certain size (Ennis et al. 1991; Mueller et al. 2016). Another important factor is the residence time within an environment conducive to aggregation. As long as growing aggregates are falling through an ash-contaminated atmosphere, or are kept in (quasi-) suspension in or above a PDC, aggregation will continue and allow thick rims to grow (e.g., Fig. 2c), or allow aggregate size to vary as a function of distance from the volcano. The largest aggregates are sometimes found at some distance from the vent (see Wallace et al. 2013; Van Eaton et al. 2015) for observations from deposits of the Redoubt event 5 in 2009, Alaska). Finally, large aggregates are, with their increased mass, subject to greater impact energies which make them prone to shattering and disaggregation (e.g., Mueller et al. 2017a). This may explain the rare occurrence of exceptionally large aggregates with diameters up to several 100% larger than the surrounding mean of aggregate diameters, both in impact and volcanic sediments.

Whether one thick rim or multiple thin rims grow around an aggregate will depend strongly on the environmental conditions and the residence time within the atmosphere. If aggregation conditions change slowly (in favor of aggregation increasing, for example, humidity or binder viscosity), a single rim could keep growing for an extended period of time. Conversely, multiple rims, and significant variation in the size of the aggregated grains, must reflect drastic changes in aggregation conditions, probably primarily controlled by the relative velocity of an aggregate and the ambient ash cloud. This may result in a change in the PSD of aggregating particles and would manifest as a second rim structure. For example, one of the aggregates from the Caldera del Rey tuff ring (Fig. 2c) shows a distinct boundary between two lithologically distinct rims, indicating the strongly changed conditions for aggregation.

We speculate that impact-related aggregates may be suspended for longer time periods within atmospheric dust clouds than, for example, volcanic aggregates. This may be due to the potentially higher initial altitudes attained by impact ejecta curtains, and this might contribute to the thicker rims on impact-generated aggregates.

### Synthesis of aggregate structures and textures from various environments

**Fig. 12:**
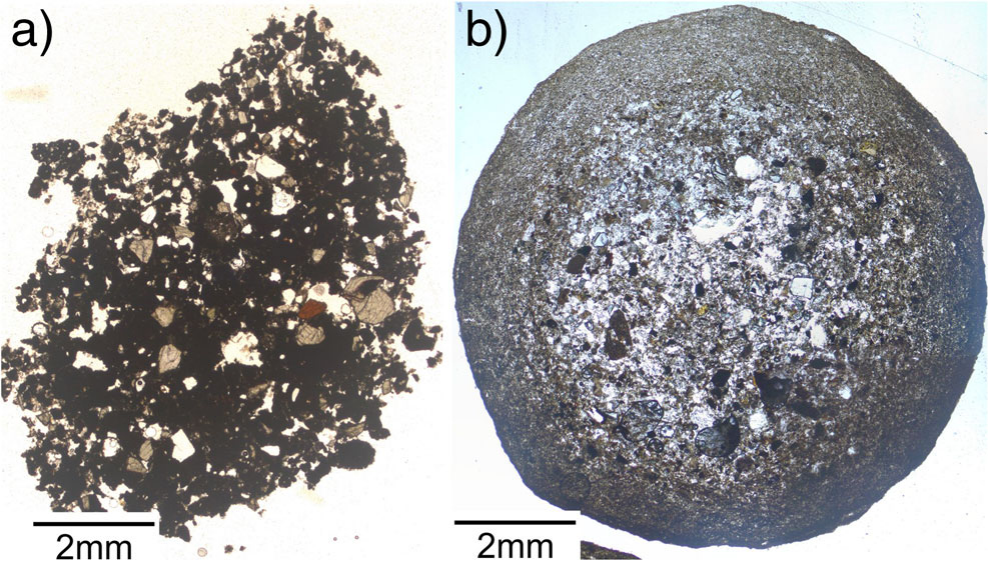
**a** Thin section of a structureless aggregate (PC1 type) from the Wehrer Kessel Volcano, Eifel. PSD is relatively confined and compared to previously described aggregates, very coarse. **b** Thin section of an aggregate sampled at Stromboli (AP2 type, Secche di Lazzaro unit). A clear distinction in core and rim is possible based on PSD

Our study reveals that aggregation is not a uniform process. Repeated aggregation and subsequent disaggregation lead to the formation of a variety of aggregate types and sizes during volcanic eruptions. Our data shows that PC and AP samples can occur next to each other in the same stratigraphic unit, and that single aggregate types are not necessarily confined to certain stratigraphic units (i.e., AP2 type samples were found both in fall and PDC deposits, see Table 1). Mueller et al. (2016) have shown that the generation of PC or AP type aggregates is strongly dependent on the PSD of the host particle cloud: very restricted initial PSDs (of just a few tens of μm diameter particles) produce PC type aggregates, whereas broad initial PSDs (of several tens to few hundreds of μm-diameter particles) tend to produce structured AP type aggregates. Samples of PC type aggregates analyzed in this study (e.g., particle clusters from Wehrer Kessel volcano, Eifel, Germany, Fig. 12a), show very narrow PSDs compared to the Stromboli AP2 type aggregates which have PSDs that range 0–500 μm (Fig. 12b). During volcanic eruptions, two sedimentation possibilities for two co-existing aggregate types (PC and AP) can be hypothesized. (1) PC1 type aggregates may in some cases represent incomplete AP2 type aggregates, which were deposited before the fine-grained rim could be (fully) established; or (2), as aggregation experiments have shown that aggregates are produced within a few seconds (e.g., Van Eaton et al. 2012; Mueller et al. 2016), areas in the plume with a more confined PSD may exist, so that during a short time window, PC1 aggregates are generated and deposited.

AP2 type aggregate formation is favored in environments with broad particle size distributions that promote selective particle aggregation processes. Whether or not a PC becomes coated with one or more fine-grained rims depends on the variability in differential velocity or vector of existing aggregate and surrounding fines. Multiple rim growth (e.g., Fig. 2e) may be favored when an aggregate is exposed to variable transport (from quasi-coupled during rise in an eruption plume to highly turbulent during fall) and ambient conditions (humidity, temperature, etc.), for example, during fallout from an ash plume and into a PDC.

The susceptibility of aggregates to erosion or destruction during transport or deposition may play a significant role in defining the stratigraphic levels in which aggregates are being found. There is a higher probability of aggregate preservation during the waning stages of PDCs (lower transport energy), and thus they may be preferentially found in the upper parts of PDC deposits. During peak intensities, aggregates that are being added from the turbulent ash cloud to more turbulent and more particle rich basal parts of PDCs, are prone to breakup or disintegrate completely (see also Brown et al. 2010, 2012). In several outcrops, the number of aggregates increases nonlinearly towards the top of individual beds (including deposits from Lago di Martigniano and Stracciacappa, Tungurahua, and Tenerife, Table 1), whereas simultaneously, the number of broken aggregates and aggregate fragments is decreasing. Similar observations have been made by Brown et al. (2010) at Tenerife, where aggregate fragments were mainly found at lower, basal parts of PDCs. The preservation potential of aggregates decreases towards the basal parts of a moving PDC due to the greater particle concentrations and increased momentum and energy of the moving material which ultimately leads to break-up and destruction of aggregates. During the waning phase of a PDC pulse, chances of survival for aggregates increase since collisions with other clasts will become both less frequent and less intense. This is manifested by the frequent upward grading of aggregate frequency in individual stratigraphic layers. A similar pattern can be inferred for the Nördlinger Ries impact deposit, where aggregates are found exclusively in discrete intervals a few cm thick in the upper parts of the crater fill ejecta, and exclusively in matrix-supported zones. In the Ries FBN 73 drill core, penetrating a total length of 1140 m of sediments, aggregates were only found at a confined vertical range of 5 m within the top part of the ejecta fall back layer. Stoeffler et al. (2013) proposed that the accretionary lapilli formed during fall from particles that had been ejected highest into the atmosphere, rich in solid dust and water vapor. Apart from the plume temperature, this model is largely similar to the volcanic scenario. It remains open if PC type aggregates have never been generated or if they have not survived transport and deposition. So far, they have not been described for the Ries or Sudbury impact sites.

Impact-generated aggregates tend to have much smaller core to rim ratios than volcanic aggregates. Only a few volcanic aggregates (e.g., the double aggregate from Tenerife, Fig. 2c) show rims with thicknesses equivalent to the respective radii of the cores. This was only reproduced by artificial means by the use of high-viscosity binders. Although aggregation in impact-generated dust clouds is analogous to aggregation in volcanic ash clouds, there clearly are differences in process that control the nature of the aggregates.

## Discussion

Throughout this study, it becomes apparent that aggregates and especially accretionary pellets from volcanic, impact, and artificial environments are nearly indistinguishable from each other; still, the respective environmental starting conditions may be drastically differing in their parameters:

- An impact event only forms one single pulse, whereas a volcanic plume can be replenished by multiple explosions during one eruption.
- The initial velocity of ejecta leaving an impact crater can be on the order of km s^−1^ (Johnson and Melosh 2012), while volcanic ash tends to have an initial velocity upon ejection from a volcano on the order of m s^−1^ (Wilson and Self 1980; Sahetapy-Engel and Harris 2009; Taddeucci et al. 2012).
- Different starting material: Impact ejecta is comprised of whatever target material is hit; in case of the Ries impact event, this includes abundant carbonates with some cherts. In case of Sudbury, this included various sediments as well as the basement granites. By contrast, volcanic lapilli are formed from clasts that derive from a largely homogeneous starting composition.
- Residence time: Because the depositional conditions of impact-generated aggregates are unknown, it is also unknown what the time of formation may have been. It is possible that impact-generated aggregates formed over periods of time that are longer than for volcanic aggregates.

Despite these clear starting differences, environmental conditions within the plume/ejecta curtain must change during later stages in order to generate such remarkably similar accretionary lapilli.

Artificial aggregation experiments have shown the preference of particles to aggregate in decelerated areas of the fluidized bed, rather than in the central, channeled, and high-velocity stream (e.g., Salman et al. 2006). Similarly, aggregation in volcanic plumes is described to be a process happening in the decelerated umbrella region or during fallout (e.g., Durant et al. 2009), rather than in the central gas thrust region of an eruptive column. Accordingly (and due to the high textural and structural similarity between volcanic and impact aggregates), we hypothesize aggregation during impact events to happen at later stages, e.g., during fallback of material. This hypothesis is supported by the occurrence of Ries aggregates at the stratigraphic top of the deposit, which is interpreted as late fallback material (e.g., Graup 1981).

The influence of the different starting materials in the particle plume seems to be of subordinate importance. Volcanic field studies and experiments have shown that preservation of aggregates is critically depending on the availability of soluble salt compounds such as NaCl or CaSO_4_ (Gilbert and Lane 1994; Mueller et al. 2017b). Volcanic eruptions offer two ways to precipitate salts on ash surfaces: (1) through precipitation of salt crystals (e.g., NaCl) out of salt rich brines (e.g., interaction of volcanic ejecta with sea water during phreatomagmatic eruptions) or (2) through diffusion-driven precipitation after the chemical interaction of acid solutions (e.g., HCl or H_2_SO_4_) with ash particles (e.g., Ayris et al. 2013, 2014). Whereas the latter mechanism may also apply for impact events, salts for binding aggregates here may further depend on the chemistry of the target material and hence significantly influence aggregation efficiency: carbonite rich cherts as in the Ries area may significantly boost the generation/presence of such salts like gypsum or calcium chloride on ejecta particles, which will in turn improve aggregate stability and their chance of preservation (Mueller et al. 2017b).

Volcanic aggregates are predominantly found in proximal to medial locations (up to a few 100 km from the source), whereas aggregates have been described in meteorite impact deposits distal (within 1000 s of km as for example, for Chicxulub, Yancey and Guillemette 2008) and medial (within 10s to 100 of km as, for example, at Stac Fada, Branney and Brown 2011) to the impact crater as well as within the impact crater itself (e.g., Sudbury, Grieve et al. 2010). The data presented here shows that volcanic and impact-generated aggregates are similar structurally and texturally, with the only discernible difference being the generally thicker rims around meteorite impact aggregates. This indicates that irrespective of the physical dynamic differences involved in the generation of the different particle clouds, there were windows of opportunity when the ambient conditions were favorable for aggregation and for the preservation of accretionary lapilli. Coupled with an understanding of the underlying physical, chemical, and mechanical aggregate formation processes derived from experiments, this provides an opportunity to better understand dust cloud processes during the deposition of impact ejecta.

## Conclusions

Particle aggregation is a common process in particle-rich environments. Upon mechanical interaction, particles stick because of liquid binding or electrostatic forces followed by mineral precipitation. Aggregates from two impact sites and 16 volcanoes have been compared to artificial aggregates generated under controlled and scaled lab experiments. During the formation of the artificial aggregates, particle properties and boundary conditions were controlled to constrain aggregation efficiency variations. All three aggregation environments produce complex, internally structured aggregates; however, unstructured particle clusters were only found in the experimental and volcanic aggregate populations. If this is due to a lack of preservation during deposition or diagenesis of impact deposits is unclear. Primary particle size distributions are similar for aggregates of all three environments. Experimental and volcanic aggregates exhibit very thin concentric rims around relatively large aggregate cores, while meteorite impact-generated aggregates commonly show rims with thicknesses that exceed the diameter of the cores—a feature that is only very rarely observed for volcanic aggregates and not at all for artificial ones. In summary, it is remarkable that meteorite impact-generated and volcanic aggregates share many similarities, and in some cases may be indistinguishable without their stratigraphic and lithological context. To date, no major impact event has been witnessed by humans; therefore, knowledge of the impact event and of particle deposition is based on interpretation of field data and on modeling. Explosive volcanic eruptions that produce aggregates have been observed and studied in greater detail, and the understanding of the underlying processes for the formation of aggregates has improved significantly in the past decades through numerical, field, and experimental studies (e.g., Costa et al. 2010; Van Eaton and Wilson 2013; Bagheri et al. 2016; Mueller et al. 2016). Based on the results of this study, we can infer that ambient conditions necessary for aggregation within dust clouds from meteorite impacts can be broadly similar to those within volcanic ash clouds and further can be reproduced in the laboratory.

## Electronic supplementary material

ESM 1 (XLSX 67 kb)
